# Molecular evaluation and genetic characterisation of Newcastle disease virus's haemagglutinin‐neuraminidase protein isolated from broiler chickens in Iran

**DOI:** 10.1002/vms3.629

**Published:** 2021-10-04

**Authors:** Mohammadreza Shafaati, Masoud Ghorbani, Minoo Mahmodi, Mostafa Ebadi, Reza Jalalirad

**Affiliations:** ^1^ Department of cellular & molecular biology, Faculty of Basic Sciences, Hamedan Branch Islamic Azad University Hamadan Iran; ^2^ Department of Research and Development Pasteur Institute of Iran, The production and Research Complex 25 Kilometer Tehran‐Kraj Highway, Karaj Alborz Iran; ^3^ Department of Biology, Faculty of Sciences, Damaghan Branch Islamic Azad University Damghan Semnan Iran

**Keywords:** broiler chicken, haemagglutinin‐neuraminidase (HN), molecular phylogenetic study, Newcastle disease virus (vNDV), virulent

## Abstract

**Background:**

Newcastle disease (ND) virus (NDV) is one of the major pathogens in poultry farms that causes severe economic damages to the poultry industry, especially broiler chicken and turkey farms. Despite the endemicity of ND and its many epidemics in the country, the nature of the Iranian strain of the Newcastle virus is still largely unknown. This study aimed to characterise and evaluate NDV isolates obtained from commercial poultry farms in Iran in 2019 through haemagglutinin‐neuraminidase (HN) gene sequencing.

**Method:**

HN gene of each NDV isolate was amplified and sequenced using specific primers followed by phylogenetic analysis of full length of HN gene open reading frame and amino acid (aa) sequence of HN.

**Results:**

Phylogenetic analysis of the HN gene showed that the virus is very closely related to genotypes VII and III. Analysis of HN gene nucleotide sequences showed that all isolates encode proteins with a length of 571 aa.

**Conclusion:**

Results of the present study are useful for a better understanding of molecular epidemiology of indigenous NDV strains and determining important molecular differences between fields and commonly used vaccine strains related to main immunogenic proteins.

## Graphical abstract

Newcastle disease (ND) virus (vNDV) is one of the major pathogens in poultries that causes severe economic damages to the poultry industry, especially chicken and turkey farms. Despite the endemicity of ND and its many epidemics in the country, the nature of the Iranian strain of the Newcastle virus is still largely unknown. This study aimed to characterise and evolute vNDV isolates obtained from commercial poultry farms in Iran in 2019 through haemagglutinin‐neuraminidase gene sequencing.

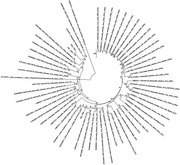

## 1 INTRODUCTION

Newcastle virus is one of the most common pathogens in birds, wreaking havoc on the poultry industry, especially poultry and turkeys (Ewies et al., 2017). The principal signs of this condition are respiratory failure, central nervous system and diarrhoea disorders (Ewies et al., 2017). The pathogenicity of the Newcastle disease (ND) virus (NDV) varies depending on virus strain and environmental conditions. Newcastle virus infection causes abrupt and asymptomatic deaths in young chickens and can be more severe with clinical signs in older birds (Ewies et al., 2017). The spread of infection occurs mostly through contact between healthy and sick birds (Suarez et al., 2020). The NDV is a family of Avaluvirus and Paramyxoviridae, which can cause highly infectious, acute ND in poultry. The virus is the most common type in poultry (Dimitrov et al., 2017; Mayo, 2002; Shafaati et al., 2013). Envelope virus with a single‐stranded, non‐segmented RNA genome of negative sense (Bello et al., 2018a; Hashemzadeh et al., 2015). The NDV genome contains six major structural genes (3′‐NP‐P‐M‐F‐HN‐L‐5′), and it has two minor proteins called W and V, which is achieved through the process of RNA editing on the *P* gene by adding guanine nucleotides (Abdisa & Tagesu, 2017; Ganar et al., 2014; Motz et al., 2013). Viral replication, transcription, translation and protein processing occur in the host cell's cytoplasm, while virus particles are assembled in the plasma membrane by budding (Bi et al., 2019). The three main pathotypes, including velogenic, mesogenic and lentogenic, of NDV are calculated based on the inclusion of the intracerebral pathogenicity index, the intervein pathogenic pathogenicity index, the mean death time and the cleavages of the F proteins (Gowthaman et al., 2016; Mayahi & Esmaelizad, 2017; Suarez et al., 2020). The two proteins, haemagglutinin‐neuraminidase (NA; HN) and F, are the main virulence factor of the virus (B. Liu et al., 2015; Ren et al., 2019). Two glycoproteins F and HN play essential roles in the assembly and development of envelop viruses and determining tropism in the host and tissues (Jin et al., 2017). HN glycoprotein has activities such as hemagglutination (HA), NA and stimulates F protein activity (Soltani et al., 2019). The binding of the HN protein to the sialic acid receptor initiates protein F action, which leads to the fusion of the virus membrane and the host cell membrane (Liu et al., 2019b). NDV virus isolates have a high mutation potential that enables several genotypes of viruses to grow at the same time. Newcastle virus strains' virulence is determined by tissue or organ tropism, the host immune system and/or replication impact (Fan et al., 2017). Full genome and structural genes for 20 Avulavirus species have been recently published in an extensive comparative study (Munir & Shabbir, 2018). The early genotypes I, II (including lentogenic viruses used to make live vaccines), III, IV and IX appeared between the 1930s and 1960s, according to NDV taxonomy. Late genotypes include NDV virus isolates discovered after the 1960s, genotypes V, VI, VII, VIII and XI. In South, Central and North America, genotype V viruses similar to late genotypes from the 1970s were isolated from poultry species and cormorants. Doves and pigeons are the most common animals with genotype VI isolates, while in the Middle East and Asia, genotype VII viruses include a broad array of hosts. The significance of this isolated community is linked to its position as poultry pathogens. Worldwide, ND outbreaks of genotype VII isolates occur. In North America, common virus genotype X (waterfowl and shorebird isolated viruses) has been identified. Genetic isolates XII NDV (poultry isolates) were found in South America and China (geese). Genotype XIII includes pathogenic isolates from Russia, Iran and Pakistan from 1995 to 2008. In West and Central Africa, genotype XIV virus isolates were found from 2006 to 2008, while genotype XV viruses were isolated from China in chickens and geese. In South and Central Africa, genotypes XVI (Dominican Republic), XVII and XVIII were also reported (Mayahi & Esmaelizad, 2017; Miller et al., 2015; Orynbayev et al., 2018). Despite the prominent role of HN glycoprotein in pathogenicity and induction of host immune responses by NDV, there is limited information on the molecular properties of this glycoprotein and its encoding gene (Soltani et al., 2019b). Currently, ND causes severe damage to the poultry industry every year. Despite the widespread vaccination of commercial poultry flocks with various active and inactive vaccines, outbreaks of the disease with different intensities are reported regularly and continuously. However, little is known about the epidemiology and molecular properties of NDV native isolates in Iran. This study aimed to characterise and evaluate ND pathogens (vNDV) isolates obtained from commercial poultry farms in Iran in 2019 through haemagglutinin‐NA (HN) gene sequencing.

## 2 MATERIALS AND METHODS

### 2.1 Virus incubation and collection

Ten tissue samples, including brain, spleen, tonsils and lungs from a poultry farm in Alborz Province with NDV mark, were received as a gift from Razi Vaccine and Serum Research Institute. Isolation of the virus according to the standard method determined by the World Organisation for Animal Health was performed by inoculating 200 μl of the suspension of the processed samples into specific pathogen‐free (SPF) 9‐day‐old egg allantoic fluid and then incubating for 96 h at 37°C in a carbon dioxide (CO_2_) incubator (OIE, 2012). After that, allantoic fluid was taken from samples that had resulted in fetal death and used for HA inhibition (HI) processing.

### 2.2 HA test

The HA assay was performed using the Alexander and Chettle (1977) method. Briefly, a total of 25‐μl phosphate buffer saline (PBS) was added to all wells of a 96‐well microtiter plate (U‐shaped well). Next, 25 μl of viral suspension was added to the first well. The virus suspension was diluted into the last well by a multichannel micropipette. In each well, a 25‐μl PBS solution was added. A total of 25 μl of 1% chicken red blood cells (RBCs) was then added to each well. The plates were then incubated at room temperature and observed after 30 min.

### 2.3 Hemagglutination inhibition (HI) test

The HI assay was used to titrate the antibody response to a viral infection using the Alexander and Chettle (1977) method. A U‐shaped base 96‐well microtiter plate was used for the HI test. Wells with a U‐shaped base, except for the first well, acted as a 4‐HA U control line. Using a multichannel pipette, 25‐μl ND‐specific antiserum was added to the first well, which was then diluted two‐fold to the next until the last wells for the titration. Then, 25 μl of 4 HA virus/antigen was added to each well. The plates were then kept at room temperature for 30 min. Alternatively, they could be placed at 4°C for 60 min. The next step was the addition of 25 μl of 0.5% random chicken RBSs into each well. The plates were then incubated for 40 min at room temperature. Agglutination patterns were checked and recorded for further evaluation.

### 2.4 RNA isolation and RT‐PCR

Viral RNAs were extracted from all HA‐positive and HI‐confirmed allantoic fluids using a High Pure Viral RNA Isolation kit (Roche) as described by the manufacturer. Complementary DNA (cDNA) synthesis was carried out by using random hexamers. With the RevertAid Reverse Transcriptase kit (Fermentas‐Thermo Fisher Scientific) in a 20‐μl reaction volume containing 1‐ng viral RNA, 60 pM final concentration of primer, 20 mM each deoxynucleotide triphosphates, 80‐U Mmulv enzyme Revertaid, 5‐U RiboluckRNase inhibitor, 4‐μl 5X reaction buffer and up to 20‐μl diethyl pyrocarbonate‐treated water. The reaction mixture was incubated for 60 min at 45°C followed by 10 min at 70°C.

Three specific primers (forward and reverse) were designed by CLC Main Workbench 4.5 (QIAGEN Co.) and used to overlap PCR products covering the entire coding area of the HN gene. In this study, three pairs of primers were designed in our laboratory based on the sequence available in the National Center for Biotechnology Information with access number KF727980 and synthesised by CinnaGen Company (Table 1).

**TABLE 1:** The sequences of primers used for amplification of haemagglutinin‐neuraminidase (HN) gene in this study

**Primer**	**Sequence (3′ to 5′)**	**Position**	**PCR product size (bp)**
HN1f	CAACAAGCACAGCAAAAGACCTTACT	6133–6162	900
HN1r	AGTATTGATGTGAATGTGAGTGA	7011–7033	
HN2f	ACGGGGCTACGAATAATAGC	6761–6780	900
HN2r	GCCTCGTTGGTACAAGAAGTG	7648–7668	
HN3f	TAATAACACATGCCCCGATG	7437–7456	877
HN3r	CGACTAAAGAAGGGACTCAGAC	8292–8312	

The PCR reaction mixture for each sample consisted 12.5‐μl master MixPCR 2x (CinnaGen), 2‐pM concentration of each primer, 2‐μl cDNA and 8.5 in a final volume of 25 μl. Amplification was programmed in a thermocycler (ABI‐USA) as follows: 94°C for 5 min followed by 35 cycles of 94°C for 30 s, 60°C for 30 s, 72°C for 30 s and a final extension at 72°C for 5 min. The amplified products were detected on SYBR Green‐stained (CinnaGen) 1% agarose gel using Tris‐Borate‐EDTA (pH 8) after electrophoresis and ultraviolet illumination. All chemicals, unless differently stated, were provided by Cinnagen. The pGET‐II cloning vector obtained from CinnaGen was used to insert all fragments.

### 2.5 Sequence determination

Recombinant vectors containing HN gene fragments for bilateral sequencing were sent to the German company MWG by an Iranian intermediary company. The results were aligned and analyzed by CLC Genomic3.6 and DNASIS MAX 3.0 (Hitachi Solutions America) and compared with selected sequences available in the GenBank. The phylogenetic tree was constructed using the maximum likelihood method based on the Tamura–Nei model (Tamura & Nei, 1993) with MEGA 7 software (Kumar et al., 2016). The evolutionary distance and homology of the respective coding region were also estimated using pairwise sequence comparison in MEGA 7 software (Kumar et al., 2016) and analyzed in Excel. Prediction of amino acid (aa) sequences and their alignments were also performed by MEGA 7 (Kumar et al., 2016).

## 3 RESULTS

### 3.1 Isolation and identification virus

**FIGURE 1:**
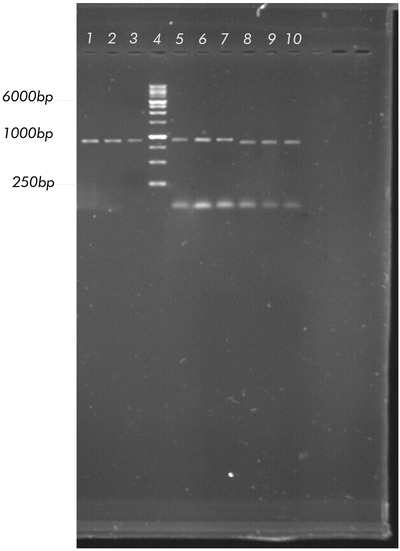
Gel electrophoresis analysis of hemagglutinin‐neuraminidase (HN) gene fragment reverse transcription polymerase chain reaction product: Lane 1–3: The amplicon HN1 primer pair (900 bp). Lane 4: 1 Kb DNA ladder (Fermentas #SM0311). Lane 5–7: The amplicon HN2 primer pair (900 bp).). Lane 8–10: The amplicon HN3 primer pair (877 bp)

NDV isolation was performed by injecting the supernatant of homogenised pulmonary and brain organs from suspected chicken samples of NDV into the allantoic fluid of SPF, embryonated and viable eggs. The NDV isolates would cause the death of chicken embryos. The allantoic fluid from the inoculated chicken eggs was then used to identify viruses. Results of HI tests and virus isolation showed that three of their HI‐test sample were positive out of 10 tissue samples. After RNA extraction and cDNA synthesis, amplification was performed by specific primers of HN gene through a polymerase chain reaction. The result for two pairs of primers is a band of 900 bp, and for the other pair of primers, the band is 877 bp on 1% agarose gel (Figure 1). Following agarose gel extraction, the fragment (lane 6 of Figure 1) was cloned into the pGET‐II cloning vector and validated by PCR analysis. The accuracy of the HN gene ORF in the pGET‐II cloning vector was confirmed by sequencing analysis using the Chromas software version 1.45 (Australia). Subsequently, (Results not presented), the sequencing data from the present study were submitted to the GenBank database with the Submission numbers of 2346063, 2396304 and 2396478.

### 3.2 Genomic features

Approximately 1980 bases of the HN gene sequence were examined in each of the three isolates. The signal for initiation of transcription of the gene start of the gene was the same in all three isolates and was determined as ACGGGTAGAA. The codons in all three sequenced HN genes have been located in the same position for both the ATG (nt92‐94; translation initiation) and TAA (nt 1805—1807; termination). The HN gene's guanine and cytosine content in isolates HN_IR1 and HN_IR2 and HN_IR3 were 46.43% and 45.78% and 48.66%, respectively.

**FIGURE 2:**
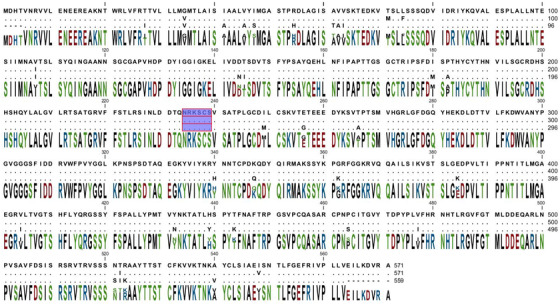
Sialic acid‐binding site: Three isolates of HN protein contained only 571 amino acid (aa) residues that were compared with Newcastle disease (ND) pathogens (vNDVs) and contained the sialic acid‐binding site (NRKSCS) at positions 234–239 (belu marker)

All three isolates of HN protein contained only 571 aa residues that were compared with vNDVs and contained the sialic acid‐binding site (NRKSCS) at the position 234–239 (Figure 2). There are three critical residues for receptor recognition at positions 401 (E), 416 (R) and 526 (Y) that in isolated three NDVs and vaccine strains has highly conserved. For the examination of antigenic sites on the HN glycoprotein, the three isolates were compared with the common vaccines of La Sota and B1 strains, showing that five aa residue substitutions at positions 347 (E to K), 494 (G to D), 514 (I to V), 521 (S to N) and 569 (D to V) has changed. These mutations may affect the antigenicity of HN protein and also among all three isolates, 13 cysteine residues at positions 123, 172, 186, 196, 238, 247, 251, 344, 455, 461, 465, 531 and 542. Among all three isolates, four conserved glycosylation sites at positions 119 (NNS), 341 (NNT), 433 (NKT) and 481 (NHT) were compared to the common strains of La Sota and B1 vaccines (Table 2; Connaris et al., 2002; X. Liu et al., 2003; Soltani et al., 2019).

**TABLE 2:** Comparison of amino acid (aa) substitutions at different positions of the deduced haemagglutinin‐neuraminidase (HN) protein sequences between three Iranian field Newcastle disease virus (NDV) isolates and two NDV vaccine strains

aa substitutions at residues																			
	Antigenic/neutralising sites																		
	Sialic acid binding site (234–239)						Residues 401, 416 and 526			Glycosylation site 119, 341, 433, 481				Residues 347, 514, 521, 569				Fusion promotion region	
Virus							401	416	526	119	341	433	481	347	514	521	569	127	145
N	R	K	S	C	S	E	R	Y	N	N	N	N	E	I	S	D	V	T
HN_IR1	.	.	.	.	.	.	.	.	.	.	.	.	.	.	V	N	V	I	.
HN_IR2	.	.	.	.	.	.	.	.	.	.	.	.	.	K	V	.	V	I	.
HN_IR3	.	.	.	.	.	.	.	.	.	.	.	.	.	.	V	N	V	I	A
B1	.	.	.	.	.	.	.	.	.	.	.	.	.	.	.	.	.	I	A
Lasota	.	.	.	.	.	.	.	.	.	.	.	.	.	.	.	.	.	I	A

*Note*: aa sequences of Iranian NDV field isolates and two NDV vaccine strains have been compared.

Abbreviations used for aas are as follows: L, leucine; S, serine; G, glycine; C, cysteine; R, arginine; E, glutamic acid; Y, tyrosine; I, isoleucine; T, threonine; V, valine; N, asparagine and K, lysine.

### 3.3 Phylogenetic analysis and distance estimation

**FIGURE 3:**
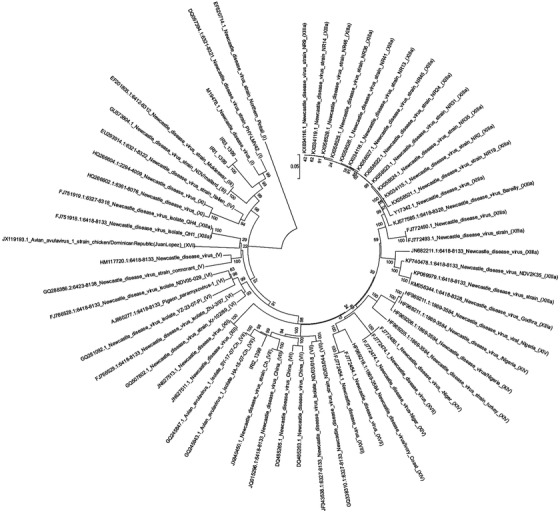
Phylogenetic analysis based on the full length of HN gene open reading frame. The evolutionary history was inferred by using the maximum likelihood method based on the Tamura–Nei model (Tamura & Nei, 1993). The tree with the highest log likelihood (‐18047.1983) is shown. Previously identified HN gene sequences of ND virus (NDV) strains representing different genotypes have been provided from the GenBank with their accession numbers. Numbers indicate the bootstrap values (1000 replicates). Horizontal distances are proportional to sequence distances. Evolutionary analyses were conducted in MEGA7 (Kumar et al., 2016)

The entire coding region of HN gene (1716 bp), IR1 and IR2 isolates analysed in the present study were closely related to genotype III and IR3 isolates. Among available sequences of HN complete coding region, a Chinese isolate (GenBank: GU573804 (IR1 and IR3) and GenBank: JX840450 (IR2)) was found to be the most similar isolate to our isolates with 99% homology. After constructing the phylogenetic tree using the complete sequence of the open reading frame (ORF) of the HN gene, the viruses representing each genotype were clearly distinguished based on the branching patterns and topology of the trees (Figure 3). In the study of the phylogenetic tree, IR1 and IR3 isolates were found to have the exact evolutionary origin. Estimates of evolutionary distances between viruses of the present study are presented in Table 3 (Le et al., 2018).

**TABLE 3:** Estimates of evolutionary divergence over sequence pairs of HN gene between three Iranian NDV field isolates and genotypes NDV

No. of base substitutions per site, SE																
		1	2	3	4	5	6	7	8	9	10	11	12	13	14	15
1	**IRI1_1399**															
2	**IRI2_1399**	0/221														
3	**IRI3_1399**	0/013	0/220													
4	**XIIIa**	0/205	0/109	0/207												
5	**I**	0/105	0/191	0/105	0/170											
6	**III**	0/132	0/190	0/133	0/166	0/086										
7	**IV**	0/132	0/160	0/136	0/130	0/092	0/077									
8	**V**	0/179	0/136	0/184	0/108	0/141	0/130	0/098								
9	**VI**	0/182	0/112	0/185	0/098	0/147	0/137	0/098	0/092							
10	**VII**	0/210	0/063	0/211	0/083	0/169	0/163	0/131	0/111	0/095						
11	**XII**	0/213	0/135	0/214	0/106	0/181	0/185	0/153	0/129	0/113	0/105	0/118				
12	**XIV**	0/219	0/145	0/218	0/111	0/191	0/177	0/155	0/137	0/122	0/110	0/131	0/132			
13	**XIIV**	0/219	0/134	0/223	0/107	0/190	0/188	0/151	0/138	0/122	0/113	0/124	0/136	0/118		
14	**XI**	0/192	0/206	0/193	0/178	0/155	0/141	0/094	0/156	0/162	0/182	0/197	0/204	0/212	0/206	
15	**XVIII**	0/226	0/137	0/225	0/113	0/189	0/190	0/157	0/133	0/123	0/109	0/127	0/132	0/122	0/119	0/206

*Note*: The number of base substitutions per site between sequences is shown. Standard error estimate(s) are shown above the diagonal. Analyses were conducted using the Tamura–Nei model (Tamura & Nei, 1993). The analysis involved 16 nucleotide sequences. Codon positions included were 1st+2nd+3rd+Noncoding. All positions containing gaps and missing data were eliminated. There were a total of 1678 positions in the final dataset. Evolutionary analyses were conducted in MEGA5 (Tamura et al., 2011).

## 4 DISCUSSION

In the global poultry industry, ND is a critical issue. Molecular epidemiology and phylogenetic analysis of NDV in Iran and Middle East countries are essential to determine the current situation and develop control measures that need to be improved (Ahmadi et al., 2016; Hassan et al., 2016). ND varies in each region according to the factors influencing the incidence and severity of the facial disease. Due to the similarity of clinical and necropsy symptoms with other viral and microbial respiratory diseases, none of the above symptoms can be considered a specific symptom (Bello, Yusoff et al., 2018a; Bello, Yusoff et al., 2018b). In the study of Rott et al., genotyping‐isolated NDV strains should be considered part of diagnostic methods in determining viral characteristics for reference laboratories, which is possible by sequencing (Bello, Yusoff et al., 2018b). In this study, the HA and HI tests were performed as conventional evaluations for NDV identification. Based on this, 10 samples of each of three positive isolates were used for the next stages of the experiment. The ability of the protein to agglutinate RBCs has been discovered. In parallel, positive results were discovered by observing the formation of RBC that precipitates at the bottom of the plate using an HI test. NDV RNA polymerase has a highly defective function. During the RNA amplification process, the occurrence of many mutations resulting from the function of this enzyme causes the emergence of new variants. Also, selection pressure is another factor influencing the formation of new variants (Soltani et al., 2019b). The HN gene has more exposure to the immune system than other NDV genes (Soltani et al., 2019b). Nucleotide mutations are more likely to occur in HN (Soltani et al., 2019b). Nucleotide mutations are highly frequent in HN, leading to the formation of new pathogenic strains of NDV, indicating the potential evolution of the Newcastle virus; therefore, this may be the reason for the persistence of ND in Iran (Dai et al., 2019; Ewies et al., 2017). Gene amplification was performed on HN fragments using primers designed in CLC Genomics Workbench 3.v. software. The presence of DNA bands showed that the nine test samples were molecularly proven to be NDV. The HN gene was completely sequenced in three isolates of the NDV to observe possible changes in antigenic epitopes of indigenous NDV and the genetic relatedness between commonly used vaccine strains and Iranian field isolates. Identity of the HN protein aa sequences among these three isolates varied from 98.9% to 100%, while the corresponding range between the Iranian field isolates and the vaccine strains was from 84.7% to 90.1%. These numbers are indicative of the notion that circulating viral strains in Alborz Province (as representative of circulating viruses in Iran) were considerably different from the vaccine strains in use and, therefore, the role of antigenic differences in weak vaccine‐induced protection can be presumed (Soltani et al., 2019; Soltani, Peighambari et al., 2019b). Also, the failure in the neutralising reaction of our field isolates of HN glycoprotein about widely used vaccine strains in Iran was the result of the substitution of specific aa at positions including 347 (E to K), 514 (I to V), 521 (S by N) and 569 (D by V). These aa substitutions may result in the disruption of antibody recognition and neutralisation capability. The results of the present study showed the findings of a recent study that showed aa translocations following incomplete sequencing of the HN gene coding region in NDV isolates isolated from different regions of Iran, compared to known lentogenic strains that were largely equal (Soltani, Peighambari et al., 2019; Soltani, Peighambari et al., 2019b). Investigators have already shown that very small changes in the nt sequence of an NDV strain may lead to prominent effects on the pathogenic specifications of the virus (Wajid et al., 2016). The lengths of the ORF of the HN gene may have various strains of NDV. Due to the appearance of translation termination codons at different locations in different gene regions, they can encode for various protein lengths between 571 and 616aa. The relationship between the length of the HN protein and the virulence of the virus recognised was demonstrated in the past (Gaikwad et al., 2016; Zhao et al., 2013). In standard avirulent or slightly virulent strains, the HN gene protein has an extended ORF of encoding longer proteins up to 616 aas residues (Soltani, Peighambari et al., 2019b). In the present study, the carboxyl‐terminal extension length and analysis of the HN glycoprotein gene did not indicate the lack of aa extension length. The length of HN glycoprotein in all three isolates was predicted to be 571 aas indicating high virulence in all viruses (Dimitrov et al., 2016). It has previously been shown that genotypes of strains III–VIII, which contain the shortest length aa (571 aa) of the glycoprotein HN, are composed exclusively of visotropic vologic strains(Dimitrov et al., 2016; Liu et al., 2019a). As a result of the high evolutionary rate of NDV strains, new genotypes have been reported in the last few decades, and many more may be identified in the future. It is believed the NDV's disease‐prone areas in endemic geographical areas are constantly under the influence of evolution and deformation factors (Samuel et al., 2013). Immuno selection pressure is another factor influencing the formation of new variants. The HN gene is more affected by the immune selection pressure than other NDV genes and results in the production of antiviral antibodies. Compared to other NDV genes under the same immune pressure, nucleotide mutations in Bashar HN are more likely to occur (Gong & Cui, 2011). Increasing the phylogenetic and antigenic distance between common vaccine strains and existing strains may lead to the formation of new pathogenic strains of NDV. Therefore, the persistence of ND in Iran may be the reason (Zhang et al., 2012). Phylogenetic analyses, of the three Iranian isolates in this study revealed that HN gene sequencing and phylogeny belonged to genotypes III (class II) and VII (class I; Miller et al., 2015; Munir & Shabbir, 2018). Genotype VII was isolated between 1997 to 2014 in Pakistan, India, Russia and Sweden. Genotypes XII, XIII and XIV have ancestors similar to genotype VII that produce different distinct lineages in the course of its evolution (Orynbayev et al., 2018; Ramey et al., 2013; Usachev et al., 2006). To date, the circulation of genotypes of NDV strains XIIIa, XIIId, VIIj and VIId in commercial poultry (Abah et al., 2020; da Silva et al., 2020), VI in domestic pigeons (da Silva et al., 2020; Rezaei Far et al., 2017) and VII in domestic poultry (Sabouri et al., 2016) have been proven in Iran. These data show that VII and XIII are among the most predominant NDV subgenotypes circulating in Iran between 1995 and 2017. Observation of genotype XIII in northern Russia increases the possibility of transmitting the virus from poultry to wild birds and the potential role of these birds in spreading the virus (Al‐Shammari et al., 2020; Tran et al., 2020). The low degree of genetic diversity between NDVs from Iran and those isolated in Russia, China and India indicates clear intra‐ and inter‐continental transmission of genotype VII strains like other genotypes throughout the named countries. The most probable cause of the distribution of NDV in the world has, in many cases, been human interventions linked to poultry and the pet‐bird trade. However, the involvement of migratory birds in NDV spread to poultry cannot be ruled out (Ramey et al., 2013). Munir et al. (2018) reported the similarity of phylogenetic analysis between HN gene and F gene in NDVs that is following our results in this study (Munir & Shabbir, 2018; Soltani, Peighambari et al., 2019). Due to the lack of sufficient knowledge about the molecular epidemiology and biological characteristics of Iranian NDVs, extensive studies on NDV isolates from poultry, live and accompanying birds in Iran seem necessary. This study and similar findings can explain the failure of vaccination programs in the country because of some aa differences in important antigenic sites of the HN in NDVs. Data obtained in our study help design new vaccines against ND.Data obtained in our study is believed to be able to help to design new vaccines against ND.

Our results are in accordance with the report from Ahmadi et al. (2010) indicating that in northwestern Iran, the presence of velogenic NDVs belonging to genotype VII has been confirmed. Genotype VII of NDV is now regarded as the major pathogen responsible for panzootic of ND. Therefore, the development and administration of new NDV vaccines that are closely related to predominant VII viruses may confer better protection than conventional vaccines.

## 5 CONCLUSION

In this study, HN gene and glycoprotein sequencing indicate significant genetic and aa differences between the studied and native NDV isolates in Iran with common vaccine strains. The present study's findings showed variations in important antigenic sites of studied NDVs isolated from different provinces of Iran that may be responsible for vaccine failure during previous years and employed. Phylogenetic affinity of the isolates studied in the present study with NDV isolated from wild birds in Russia and isolates obtained from China probably indicate the prominent role of migratory wild birds and trade in the widespread NDV strains and the importance of measures. Moreover, the present results may also indicate the need for designing and producing new efficient NDV vaccines in Iran.

## AUTHOR CONTRIBUTION

Mohammadreza Shafaati: Investigation, Project administration; Masoud Ghorbani: Project administration, Supervision; Minoo Mahmoodi: Supervision; Mostafa Ebadi: Data curation, Writing‐review & editing; Reza Jalalirad: Data curation, Writing‐original draft.

## CONFLICT OF INTEREST

All the authors of the manuscript declare no scientific and financial conflict of interests.

## ETHICS STATEMENT

The authors declare human ethics approval was not needed for this study.

### PEER REVIEW

The peer review history for this article is available at https://publons.com/publon/10.1002/vms3.629

## AUTHOR CONTRIBUTIONS

Mohammadreza Shafaati participated in designing the study, carried out all the experiments, compiled and interpreted the data and drafted the manuscript. Masoud Ghorbani managed the whole research project as the principal investigator and supported in drafting and also helped in the final revision of the manuscript. Minoo Mahmodi coordinated the laboratory activity and assisted in the experiments. Mostafa Ebadi and Reza Jalalirad facilitated in providing all kinds of instrumental access and management. Also, Mostafa Ebadi was involved in statistical analysis. The authors have read and approved the final version of the manuscript.

## Data Availability

The data that support the findings of this study are available from the corresponding author upon reasonable request.
